# Comparing two intramedullary devices for treating trochanteric fractures: A prospective study

**DOI:** 10.1186/1749-799X-5-9

**Published:** 2010-02-18

**Authors:** Konstantinos G Makridis, Vasileios Georgaklis, Miltiadis Georgoussis, Vasileios Mandalos, Vasileios Kontogeorgakos, Leonidas Badras

**Affiliations:** 1 Orthopaedic Surgeon, Resident, Department of Orthopaedic Surgery, General Hospital of Volos, Polimeri 134, 38222, Greece

## Abstract

**Background:**

Intertrochanteric fractures are surgically treated by using different methods and implants. The optional type of surgical stabilization is still under debate. However, between devices with the same philosophy, different design characteristics may substantially influence fracture healing. This is a prospective study comparing the complication and final functional outcome of two intramedullary devices, the intramedullary hip screw (IMHS) and the ENDOVIS nail.

**Materials and methods:**

Two hundred fifteen patients were randomized on admission in two treatment groups. Epidemiology features and functional status was similar between two treatment groups. Fracture stability was assessed according to the Evan's classification. One hundred ten patients were treated with IMHS and 105 with ENDOVIS nail.

**Results:**

There were no significant statistical differences between the two groups regarding blood loss, transfusion requirements and mortality rate. In contrast, the number of total complications was significantly higher in the ENDOVIS nail group. Moreover, the overall functional and walking competence was superior in the patients treated with the IMHS nail.

**Conclusions:**

These results indicate that the choice of the proper implant plays probably an important role in the final outcome of surgical treatment of intertrochanteric fractures. IMHS nail allows for accurate surgical technique, for both static and dynamic compression and high rotational stability. IMHS nail proved more reliable in our study regarding nail insertion and overall uncomplicated outcome.

## Introduction

Pertrochanteric fractures constitute one of the commonest fractures of the hip. They mainly occur in elderly people due to osteoporosis. Their incidence will probably continue to increase in the near future because of population aging [1,2]. The goal of treatment is fracture reduction and stable osteosynthesis to allow immediate mobilization. For many years, the sliding hip screw and plate had been the gold standard in treating pertrochanteric fractures [3-5]. Nowadays, there is an increasing interest in intramedullary nailing, especially for the unstable pertrochanteric fractures. There are several studies comparing intramedullary hip screw (IMHS, Smith & Nephew) to other intramedullary devices or sliding hip screw [6-8]. No data are available in the literature about the ENDOVIS (Citieffe) nail. No study has prospectively compared the IMHS to the ENDOVIS nail, specifically in the unstable fracture patterns.

This is a prospective randomized study in order to compare the clinical results of these two intramedullary devices, which have different design characteristics.

## Patients and methods

Between July 2005 and June 2007, 261 consecutive patients who sustained a pertrochanteric fracture were operated. Inclusion criteria for the study were patients over 60 years old with a pertrochanteric fracture after a fall that was considered low energy injury. Forty six patients with pathologic fractures, or a high energy injury and patients under 60 years old were excluded. In 110 patients it was used the IMHS and in 105 the ENDOVIS nail. The patients were randomly dispersed to one of the two treatment options by the use of sealed envelopes containing cards, indicating the treatment for each patient.

In the IMHS treatment group, 34 were men and 76 women. In the ENDOVIS group there were 33 men and 72 women. The mean age was 83.5 years (range 69-95 years) in the IMHS group and 83.9 years (range 71-96 years) in the ENDOVIS group.

Fracture stability was assessed according to the Evan's classification as modified by Jensen [9,10]. Thirty seven fractures was graded as stable and 73 as unstable for the IMHS while 39 as stable and 66 as unstable fractures for the ENDOVIS group (Table 1).

**Table 1:** Patient's and fractures characteristics

	IMHS	ENDOVIS
Number of patients	110	105
Men	34	33
Women	76	72
Age	83.5 (69-95)	83.9(71-96)
Stable fractures	37	39
Unstable fractures	73	66

Prophylactic intravenous second generation cephalosporin was administered before operation and discontinued 48 hours postoperatively. Patients were mobilized on second post-operative day, allowing them to bear weight as much as they could tolerate. All cases received anticoagulant prophylactic therapy with low molecular weight heparin, starting on admission and for 4 weeks postoperatively.

Data recorded in all patients and included the type of the fracture, the preoperative blood hemoglobin level and walking ability before fracture (Table 2). The operative data were surgical time, blood loss and any intraoperative complication. Postoperatively, the level of hemoglobin was recorded on the first postoperative day, the mobility status at the time of discharge, the duration of hospital stay and the mortality rate at 12 months.

**Table 2:** Patients' preoperative walking ability

	IMHS	ENDOVIS
Independence walking	62 (56.4%)	64 (61%)
Assisted walking	45 (41%)	37 (36%)
Bedridden	3 (3.6%)	4 (3%)

The patients were evaluated for their functional status and by serial plain radiographs at 1, 3, 6 and 12 months after operation. Fracture healing was judged based on increased sclerosis and obliteration of fracture lines. X-rays interpreted in association with clinical data and more specifically by the elimination of pain during weight bearing. In order to estimate the functional outcome the Parker-Palmer mobility score was used [11].

### Implant description

IMHS features a cannulated intramedullary nail with a 4 degrees mediolateral bend to allow for insertion through the greater trochanter. The nail is used with a standard AMBI/CLASSIC lag screw, compression screw and 4.5 mm locking screws. A sleeve, which is held by a set screw, passes through the nail and over the lag screw. The sleeve helps prevent rotation, while allowing the lag screw to slide. Standard IMHS is available in two angles (130-135 degrees), in four distal diameters (10, 12, 14, 16 mm) with a proximal diameter of 17.5 mm. Its length is 21 cm.

ENDOVIS is made of titanium alloy with a cervico-diaphyseal angle 130 degrees, a metaphyseal angle 5 degrees and total length 195 mm. The diameter proximally is 13 mm and distally 10 mm. There are two holes for cephalic screw insertion and one for the distal screw. The cephalic screws are available in nine length sizes, 7.5 mm diameter, self-drilling and self-taping. The distal screw is available in four sizes, 5 mm diameter, self-drilling and self-taping. The distal tip of the nail has a diapason section.

Operations were performed on a fracture table under spinal anesthesia and image intensifier control. After closed reduction of the fracture, a longitudinal incision started proximal to the greater trochanter apex and extended proximally about 4-10 cm, depending on the size or obesity of each patient. After splitting the aponeurosis, the entry point was made just on the tip of the greater trochanter. The nail was inserted into the femur diaphysis without reaming. Our goal was to insert the hip screw under the midline of the femoral neck, advancing the tip of the screw close to the subarticular surface of the femoral head. Tip to Apex Distance (TAD) was measured from the tip of the guide wire. When TAD value was less than 25 mm, we proceeded to reaming and insertion of the cephalic screw. Fluoroscopic control was performed to ensure that joint line was not penetrated after screw placement. Distal locking was made preferably with 2 screws.

### Statistical analysis

All data were recorded and statistically analyzed. Pearson chi-square test, Fisher's exact test and Student t-test were performed to discriminate differences between the 2 groups. Significance levels were set at *P *< 0.05. All tests were calculated using the SPSS, version 13.0 (SPSS Inc., Chicago, IL, USA) statistic package for personal computers.

## Results

The mean time needed for the two intramedullary nails procedures was 25.4 minutes (range 17-45 min) in IMHS group and 24.8 minutes (range 21-51 min) in ENDOVIS group. As expected, there were no significant statistically differences between the two groups regarding blood loss and transfusion requirements (Table 3).

**Table 3:** Preoperative and postoperative Hb level and transfusion requirements

	IMHS	ENDOVIS
Hb preoperative	11.7(8.75-14.3)	11.3(8.69-14.5)
Hb 1^st ^postoperative day	9.97(8.09-12.8)	9.85(8.15-12.65)
Transfusions IU/patient	1.73	1.8
Patients transfused	26.2%	26.6%

In IMHS group 35 (31.8%) patients achieved independent walking, 57 (51.8%) patients needed a walking aid and 18 (16.4%) were not able to ambulate. The corresponding values in the ENDOVIS group were 28 (26.7%), 48 (45.7%), 29 (27.6%) (Table 4). The mean preoperative Parker-Palmer mobility score was 7.27 for IMHS group and 7.23 for ENDOVIS group. The mean postoperative Parker-Palmer mobility score was 6.4 for IMHS and 4.7 for ENDOVIS. Statistical analysis between the 2 treatment groups revealed significant difference, favoring the IMHS treated patients (Chi-square test, p < 0.05).

**Table 4:** Patients' postoperative walking ability

	IMHS	ENDOVIS
Independent walking	35 (31.8%)	28 (26.7%)
Assisted walking	57 (51.8%)	48 (45.7%)
Bedridden	18 (16.4%)	29 (27.6%)

Two patients from the IMHS group and three from the ENDOVIS died during the hospital stay. The overall mortality rates at one year were 15.45% and 15.23% respectively with no statistical difference observed between the two study groups.

The standard length size of these two nails was used in all patients. In 8 cases the proximal sliding screws were misplaced and in 2 the proximal holes were completely missed in the ENDOVIS group. Additionally there was proximal screws back-out in 5 patients and screw joint penetration in 3 patients. Only one proximal lag screw was misplaced by using IMHS nail with no cases of back-out or screw joint penetration.

Distal locking screws were missed in 5 patients; there were 4 cases in ENDOVIS group and 1 case in IMHS group. Moreover, 5 patients treated with ENDOVIS nail underwent medial displacement of the femur diaphysis with a consequent shortening of the affected femur. No case of this complication existed in patients treated with IMHS (Table 5).

**Table 5:** Complications of 215 patients treated for trochanteric fracture

	IMHS	ENDOVIS
Missing of proximal hole	0	2
Misplaced proximal screws	1	8
Failure of distal locking	1	4
Femoral shaft medialization	0	5
Femoral shaft fracture	1	0
Cut out	1	3
Z -phenomenon	0	1
Reverse Z phenomenon	0	1
Proximal screws back-out	0	5
Joint penetration	0	3
Periprosthetic fracture	1	0
Nail breakage	1	1
Infection	2	2
		
No. complications	8	35
Percentage	7.3%	33.4%

In 4 cases cut-out was observed, associated with malposition of the proximal lag screws, three of them occurred in the ENDOVIS nail. All these cases were treated with reoperation using the IMHS nail, without any further complications.

There was one case with Z phenomenon and another one with reverse Z phenomenon treated with the ENDOVIS. These 2 complications occurred within the first two months and treated by replacing the nails with another ENDOVIS.

One intra-operative fracture of femoral diaphysis occurred in IMHS group in a patient with narrow medullary canal. This fracture treated with circular wires and healed uneventfully.

**Figure 1:**
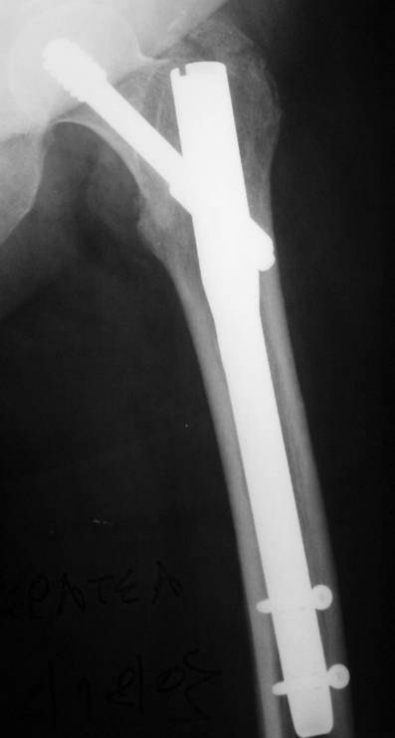
**Pertrochanteric fracture treated with IMHS nail**.

**Figure 2:**
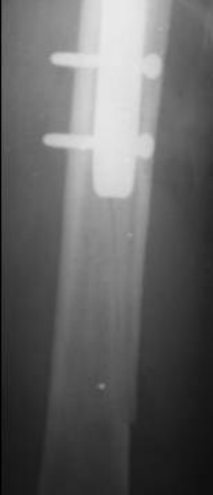
**Periprosthetic fracture at the distal tip of the IMHS three months postoperatively**.

On postoperative month three, 1 periprosthetic fracture occurred at the distal tip of the IMHS as a result of a simple fall of the patient on the ground (Fig. 1, 2). This fracture treated successfully with bone grafting and circular wires.

Two nails broke one in each group, at the site of insertion of the proximal lag screws, without necessitating further treatment.

Two cases of superficial soft tissue infections occurred in each group and were treated successfully with intravenous antibiotic administration after culture and isolation of the specific pathogens.

All types of complications in association to type of fracture (stable vs. unstable) are shown on Table 6. The overall complication rate was higher for the unstable fractures in both groups.

**Table 6:** Complications in relation to the fracture type

	IMHS		ENDOVIS	
	Stable	Unstable	Stable	Unstable
Missing of proximal hole	0	0	1	1
Misplacement of proximal screws	0	1	4	4
Failure of distal locking	0	1	3	1
Femoral shaft medialization	0	0	0	5
Femoral shaft fracture	1	0	0	0
Cut out	0	1	1	2
Z -phenomenon	0	0	1	0
Reverse Z phenomenon	0	0	0	1
Proximal screws back-out	0	0	2	3
Joint penetration	0	0	2	1
Periprosthetic fract	1	0	0	0
Nail breakage	1	0	0	1
Infection	1	1	1	1

All fractures considered healed clinically within 8 weeks in all patients, with the exception of those with the mechanical failure who needed reoperation.

## Discussion

The ideal implant for stabilization of pertrochanteric fractures is still under debate. Many authors consider the sliding hip screw with a plate the best choice, extenuating its favorable results, the low rate of hardware failure and non-union. A recent metaanalysis compared the sliding screw and plate with intramedullary nails (IMN) [12]. Total fixation failure rate was higher in the IMN group, without reaching statistical significance. However, intramedullary nails gain a continuous popularity for both stable and unstable fractures, due to certain theoretical advantages and ease surgical technique. Additionally, the small incisions result in less blood loss intraoperatively. A variety of intramedullary devices have been used with different design characteristics [13-15]. However, the adequacy and stability of fixation plays an important role, determing the success of the surgical treatment of pertrochanteric fractures [16].

**Figure 3:**
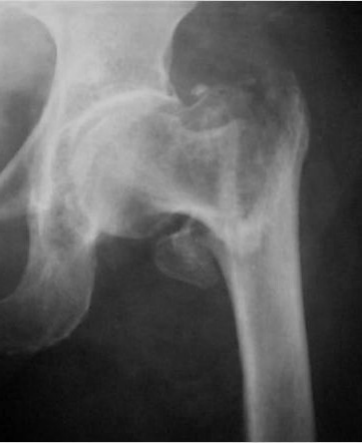
**Comminuted unstable pertrochanteric fracture treated with ENDOVIS nail**.

**Figure 4:**
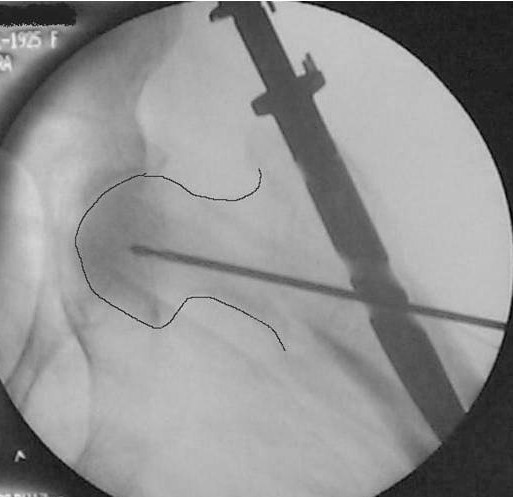
**Fracture alignment, with restoration of cervical-diaphyseal angle and anteversion is achieved by closed means**.

**Figure 5:**
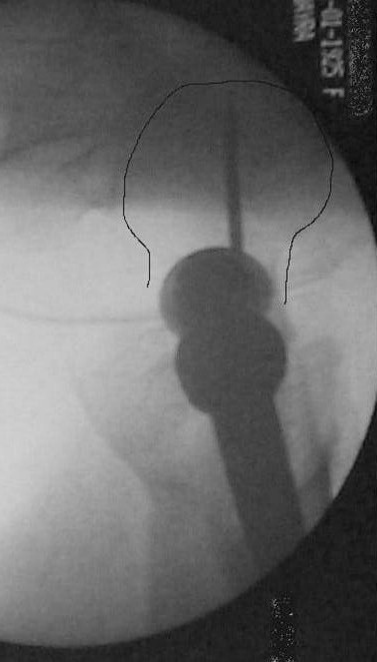
**Guide wire, for screw reaming, is inserted just bellow midline in AP, close to the articular surface**.

**Figure 6:**
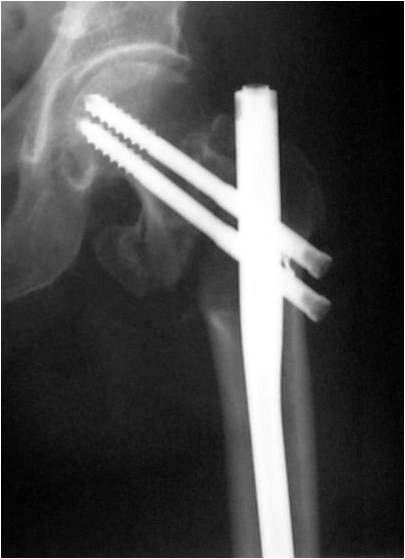
**Guide wire, for screw reaming, is inserted in the midline in lateral view, close to the articular surface**.

**Figure 7:**
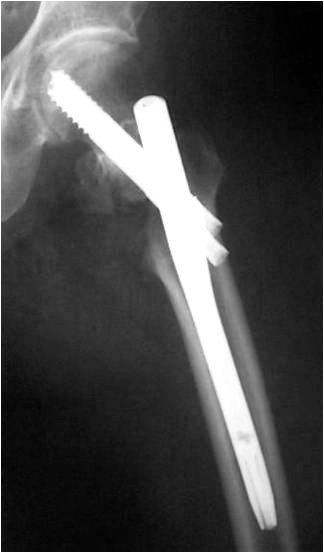
**At final x-rays, the 2 proximal screws were inserted slightly convergent and retroverted**. The femoral head reduced in slight valgus and gap at the medial site of the fracture is noticed at final x-rays.

The right position of the lag screw near the centre of the femoral head and neck, in both anteroposterior and lateral views, is critical and has been emphasized by many authors. Baumgartner et al [17] indicated the significance of tip-apex distance value in the placement of the proximal lag screw and Den Hartog [18] showed that this optimal position prevents the rotation of the femoral head and neck during the lag screw insertion. In our series, although initial drill guides were placed in an optimal position according to intra-operative TAD value measurements, the appropriate position of the cephalic screw was better achieved with IMHS nail (Fig. 3, 4, 5, 6, 7). Probably this is attributed to the cannulated screw design. In contrast, the compact form of ENDOVIS cephalic screws resulted in a significant number of screw malposition associated with increased cases with screw cut-out. When we compared the failure rate (in each treatment group) with the fracture stability (stable vs. unstable), no association with type of fracture was detected.

**Figure 8:**
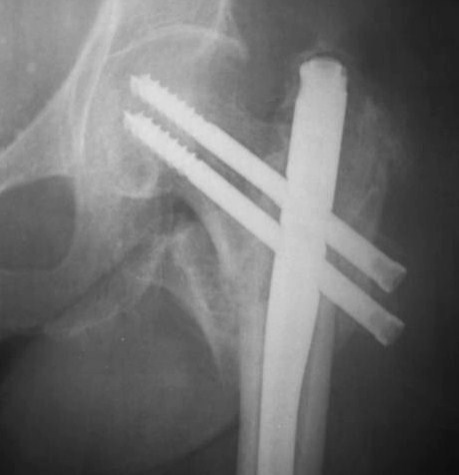
**Pertrochanteric fracture treated with ENDOVIS nail**.

**Figure 9:**
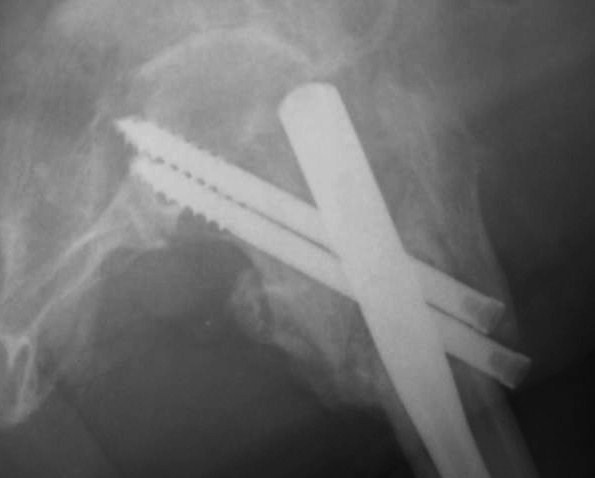
**Impaction of the fracture during weight bearing resulted in screw joint penetration three months postoperatively**.

Controlled fracture impaction and axial loading are of significant importance especially in the unstable pertrochanteric fractures [19,20]. These factors allow direct contact between the fracture fragments; promote healing, decrease the moment arm and the stresses on the implant. Compression at the fracture interface can be done intra-operatively by tightening the compression screw, adding stability to the bone-hardware construct. ENDOVIS doesn't provide the ability for intra-operative compression. Compression occurs during the healing process, under fracture loading. However, this phenomenon was not controlled and cephalic screws back-out or joint penetration was noticed in 8 cases, although initial screw placement in the femoral head was considered optimal (Fig. 8, 9). In contrast no such complication was noticed in the IMHS group.

The frequency of Z-effect and reverse Z-effect is not negligible and it has been reported by several orthopaedic surgeons using trochanteric intramedullary rods which possess two proximal lag screws [21-23]. In our series the use of ENDOVIS nail stressed these complications and resulted in an increased number of revisions. In contrast, the single femoral head screw of IMHS eliminates these complications and moreover provides an ease and safe solution, particularly in narrow femoral necks, where the positioning of two cephalic lag screws is not always feasible.

Lindsey and Rosson [24,25] have pointed out the difficulty for secure placement of the distal locking screws. Any error may result in the drilling of unnecessary holes and creates an additional stress riser that influences the bone mechanical properties. Lacroix [26] stated that distal screws should be used only when the fractures requires an extra stability. In our series failure of ENDOVIS distal locking had the result of an increased number of femoral shortening and rotational instability. The great number of distal screws misplacement is probably due to ENDOVIS small diameter. These features caused an eccentric position of the nail, mainly in wide medullary canals and directed the tip of the drill out of the distal hole. On the other side, IMHS has a more compact form and provides more diameter options. Thus, not only secures the femoral distal locking but also retains the fracture's rotational stability even if the distal locking fails.

A femoral shaft fracture during intramedullary nailing or postoperatively is a common complication [27]. In this study there was such a fracture only with the use of IMHS nail. Regarding the size of the nail, we commonly used 10 mm diameter nails. In cases with much widened diaphyses secondary to senile osteoporosis (as was the vast majority of our patient, mean age >80 years old), we easily inserted unreamed nails with a 10 mm or larger diameter. This explains why we had only one intra-op diaphyseal fracture in the IMHS group, in a patient with a narrow medullary canal.

The ambulatory status after an operation for an pertrochanteric fracture depends on different factors [28-30]. Specific parameters such as the patients' preoperative walking capability, their medical condition and comorbidities were similar to both groups. The overall walking competence in patients treated with IMHS was superior to ENDOVIS group which was statistically significant. The favorable results of IMHS group are probably explained by design differences. It seems that IMHS allows for a more accurate nail placement, secure and stable fixation with lesser complications and failures. Subsequently this is reflected to the greater walking independence of the patients and their advanced rehabilitation.

Devices combining the general principles of the sliding hip screw with an intramedullary nail constitute a safe and accurate mode of fixation for pertrochanteric fractures. Certainly, further investigations are necessary in order to prove the ideal treatment method for these fractures. However, this study indicates the IMHS device as suitable for the treatment of stable pertrochanteric fractures, those with reverse obliquity, comminuted fractures and those with a subtrochanteric extension. The features of the implant and the instrumentation for screws and nail insertion, allows for accurate and ease fracture fixation with a low rate of complications.

## Competing interests

The authors declare that they have no competing interests.

## Authors' contributions

KM carried out the data collection, participated in the design of the study and drafted the manuscript. VG participated in the data collection. MG performed the statistical analysis. VM carried out the collection and the elaboration of the images. VK participated in the design of the study and its coordination. LB conceived of the study and participated in its design and coordination. All authors read and approved the final manuscript.
